# Broiler Chicks’ Motivation for Different Wood Beddings and Amounts of Soiling

**DOI:** 10.3390/ani10061039

**Published:** 2020-06-16

**Authors:** Valerie Monckton, Nienke van Staaveren, Alexandra Harlander-Matauschek

**Affiliations:** Department of Animal Biosciences, University of Guelph, 50 Stone Road E., Guelph, ON N1G 2W1, Canada; vmonckto@uoguelph.ca (V.M.); nvanstaa@uoguelph.ca (N.v.S.)

**Keywords:** welfare, maximum price, chicken, *Gallus gallus domesticus*, bedding, litter, preference, motivation, operant methods

## Abstract

**Simple Summary:**

Many animals move excreta—or feces—away from resting areas to avoid attracting predators and spreading disease. However, today’s farms raise broiler (meat) chickens in large barns with stocking densities that prevent the birds from segregating their excreta. Moreover, whether or not chickens would prefer to avoid their excreta is unknown. Understanding what litter conditions chickens prefer can help inform farming practices. Therefore, this experiment aimed to assess chicks’ motivation to access unsoiled bedding or soiled litter. We used six pens of six to seven broiler chicks—each pen divided into two compartments by a barrier containing two one-way push-doors. The ‘home’ compartment contained soiled wood shavings, while the ‘treatment’ (T) compartment contained either aspen wood shavings, pine and spruce wood shavings, soiled pine and spruce wood shavings, ammonia reductant treated soiled pine and spruce wood shavings, or a feed treatment as a gold standard. To determine the chicks’ motivation to access the resources, the door leading into T weighed 0% (lifted), 10%, 20%, or 30% of the chicks’ body weight. The combination of time spent in T, number of visits to T, and average maximum weight pushed to access T were used to measure motivation. Chicks showed equal motivation for all substrates and preferred feed over all substrates. However, future experiments must explore chicks’ preference and motivation over the long-term in commercial conditions.

**Abstract:**

In the wild, excreta soiled surroundings can attract predators and spread disease. Yet, farmers rear broiler chicks in large barns with stocking densities that prevent excreta segregation. To measure chicks’ motivation to access unsoiled bedding or soiled litter (collectively, substrates) we used 40 16-day-old broiler chicks who were divided into six two-compartment pens. The ‘home’ compartment (H) contained soiled wood shavings, while the ‘treatment’ compartment (T) contained either aspen wood shavings, pine and spruce wood shavings, soiled pine and spruce wood shavings, ammonia reductant treated soiled pine and spruce wood shavings, or a feed treatment as a gold standard. The barrier separating the compartments had two one-way push-doors that chicks pushed to access a resource. The chicks’ motivation was measured by the average maximum weight pushed to access each resource. The door leading to T weighed 0% (raised), 10%, 20%, or 30% of the chicks’ body weight, and chicks could return to H via a raised (for 0%) or unweighted door. Our findings indicate that chicks worked hardest for feed, but paid a lower, equal price to access all substrates. With increasing door weight, chicks visited less and spent less time with the substrates. Therefore, as chicks themselves do not avoid litter that could have potential negative effects on their well-being, it is important that farmers diligently monitor litter conditions as their primary care-takers.

## 1. Introduction

Many animals, including insects, mammals, and birds, defecate and urinate away from resting and nesting sites [1,2,3,4]. Doing so not only reduces the risk of disease [1,3], but also prevents drawing in predators [5]. As a result, many animals choose to forage away from excreta or feces [6], while some species of birds go so far as to produce fecal sacs that their parents can remove from the nest [4]. 

Given the evolutionary importance of excreta segregation, logic would dictate that domestic birds, such as chickens bred for meat production (broilers), would prefer to avoid soiled environments. Commercial broilers are kept in a single open space with thousands of birds, which can lead excreta to rapidly accrue on bedding during the chicks’ ≈42 day lives. This stocking density limits birds’ ability to separate space into clean and excreta soiled areas [7,8]. Standard recommendations for litter (accumulated bedding, feathers, excreta, and waste feed) do not go beyond requiring that litter not be toxic to birds and that it be maintained at an adequate moisture level [7]. Therefore, animals that are motivated but unable to move away from excreta soiled areas may feel frustrated [2]. Additionally, soiled litter may inhibit effective preening and dustbathing to clean feathers, which may also lead to frustration [9].

Besides the possible frustrations of being unable to escape a soiled environment and developing unclean integument [10], inadequately managed soiled litter may lead to health concerns. For instance, moist litter increases the risk of contact dermatitis [11,12,13] and increases ammonia production from litter [14,15] that can cause respiratory and eye disease [16]. However, wood-based bedding material alone can cause adverse health effects, including respiratory disease [17,18,19] and dermatitis [20,21] in humans. As well, rodent studies have shown that wood-based beddings from softwoods (e.g., pine and spruce) are strongly cytotoxic, while hardwoods (e.g., aspen) tend to be much less cytotoxic [22] but not as inert as non-wood beddings like corn-cob [23].

Yet, while poultry prefer less ammoniated environments [24,25], they have also been reported to forage in [26] and consume excreta [27] in the absence of other foraging substrates. Although excreta is not a preferred foraging substrate, chickens kept on bedding will consume 5–24% of excreta produced by their group in a behavior known as social homocoprophagy [28]. One of the possible functions of social homocoprophagy may be to use the vitamins, fats, and proteins contained in cecal excreta. Therefore, this inclination may indicate that, despite increased ammonia concentrations in soiled environments, domestic chickens may be indifferent to soiling. Theoretically then, ammonia reductants could assess birds’ relative preference for soiled litter with reduced ammonia. However, these chemical ammonia reductants act by acidifying litter, and may, therefore, be aversive themselves [29].

Preference tests offer animals the opportunity to show their relative preferences for a set number of choices ranging from different floor types [30] to colors of light [31]. However, preference tests do not show strength of motivation. To solve this, consumer-demand motivation tests present animals with increasingly challenging obstacles that they must overcome to access a resource; for example, pecking a key [32], overcoming a barrier [33], or pushing a weighted door [34]. Hence, our experiment aimed to assess broiler chicks’ motivation to access four different substrates (fresh pine and spruce wood shavings, fresh aspen wood shavings, soiled pine and spruce wood shavings, and soiled pine and spruce wood shavings treated with an ammonia reductant) compared to their motivation to access feed using weighed push-doors. Furthermore, we used the average maximum weight pushed—or maximum price paid—as a measure of motivation [35,36]. This method measures the value of a resource to an animal by evaluating how much work the animal will do to access said resource [37]. The maximum price paid for the substrates was then compared to broiler chicks’ motivation to access feed, the gold standard of comparison [32]. Additionally, the chicks’ ability to leave the treatment substrate without paying a cost meant that the reward size (length of visits) was under the chicks’ control. Therefore, we also recorded the time spent and the number of visits to each treatment with varying price. This knowledge would help increase our understanding of what litter conditions chickens prefer and ultimately can help inform farming practices.

Due to the differences in human adverse reactions and cytotoxicity in rodents, we predicted that the chicks would show greater motivation to access fresh aspen over fresh pine and spruce shavings. We also predicted that the chicks would be less motivated to access soiled shavings compared to substrates with reduced ammonia. Thus, we predicted that the chicks would differentiate between the substrates in the following rank order, from most to least preferred: fresh aspen wood shavings, ammonia reductant treated soiled wood shavings, fresh pine and spruce wood shavings, and soiled wood shavings.

## 2. Materials and Methods

### 2.1. Ethical Approval

The University of Guelph Animal Care Committee (Animal Utilization Protocol Number 4105) approved this study before testing. Additionally, we followed the ARRIVE guidelines in the planning and conducting of this experiment [38].

### 2.2. Housing, Feeding, and Management

We raised 40 Ross 708 female broiler chicks in two 116 × 116 cm floor pens with 20 chicks/pen until they reached 2 weeks of age. For the first 2 weeks of life, the chicks were placed on a mixture of Pestell™ pine and spruce and Grreat Choice^®^ aspen wood shavings that were allowed to build up with excreta. Each pen was provided with a heat lamp, heat mat, and brooder (darkened) area. Chicks were provided with ad libitum feed and water, and with natural light through windows. The temperature was monitored and maintained according to the Ross 708 management guidelines [39], and the chicks were provided with commercial feed throughout the experiment.

**Figure 1:**
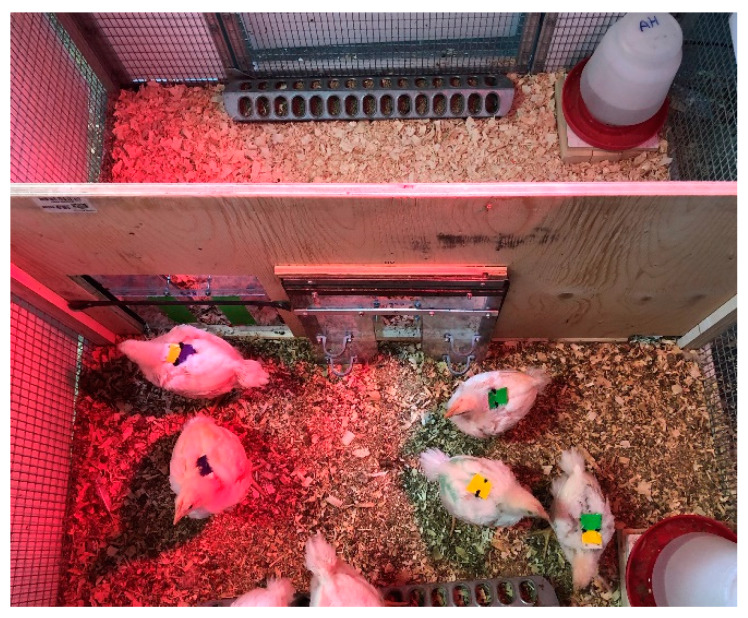
Image of broiler chick floor pens with heat lamps on. Each pen contained an H (**bottom**) and T (**top**) compartment separated by a wooden barrier with two unidirectional polycarbonate push doors. H and T alternated sides daily. The unidirectional push doors are lowered in this image. Weights could be added to the doors, which was only done for the door leading to the T compartment. Water and feed were available in both compartments except during the feed treatment.

When the chicks were 16 days old, we divided the birds of each of the two floors pens across a final six floor pens into groups of six to seven birds per pen. Birds that were kept within one pen during the first 2 weeks of life were kept together (i.e., no mixing of birds between the two original floor pens). These 116 × 116 cm floor pens contained heat mats and heat lamps until the chicks reached approximately 4 weeks old. We differentiated individual birds in each pen using colored backpacks made of colored tape, hair elastics, and soft foam (Figure 1). We checked the backpacks’ fit every three days. Within a week of the experiment’s conclusion, all 39 birds were adopted by sanctuaries and homes.

### 2.3. Substrates

At 9 and 10 days old, the chicks were habituated to the substrates: fresh pine and spruce wood shavings (FP), fresh aspen wood shavings (FA), soiled pine and spruce wood shavings taken from other broiler chick pens (SP), and soiled pine and spruce wood shavings treated with an ammonia reductant (37 kg/100 m^2^ of PLT^®^—Poultry Litter Treatment, Jones-Hamilton Co., OH, USA) (TSP). The soiled substrates (SP and TSP) were soiled shavings obtained from other broilers (2 weeks of age) kept under standard conditions at the research facility. The SP litter functioned as a negative control. All four litters were placed in the corners of chicks’ pens to habituate the chicks and during this time the litters could be freely explored for 24 h.

**Figure 2:**
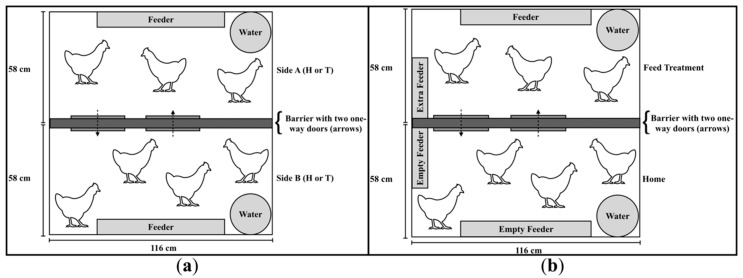
Experimental setup of broiler chick floor pens. Each pen contained two compartments (H and T) separated by a barrier with two unidirectional push doors that could be loaded to 10%, 20%, or 30% of the pen’s median chick body weight. T and H alternated between Side B or Side A to avoid side bias. (**a**) Pen setup when testing substrates (soiled pine and spruce shavings, soiled pine and spruce shavings treated with an ammonia reductant, fresh pine and spruce shavings, and fresh aspen shavings). (**b**) Pen setup when testing feed treatment.

At 16 days, birds were moved to one of the six experimental pens that comprised two compartments that alternated sides daily and had ad libitum access to feed and water. The home compartment (H) contained litter from the original two rearing pens (first 2 weeks of life) at a litter depth of 4 cm (Figure 2a). The treatment compartment (T) contained one of the four aforementioned litter types. Litter depth in T was kept the same as in H, namely 4 cm. In addition to these four substrates, we included a feed treatment as a gold standard (positive control) [32]. In this treatment, soiled shavings were provided in T and H and the feeder in H was removed—forcing the chicks to move to T to access feed. This treatment was the only time that feed was not accessible in H (Figure 2b). 

Litter samples (500 g) were collected at the start and the end of the experiment (home litter, SP), at the beginning and end of the projected chemical saturation point (7 days) for TSP, and once for the fresh shavings as these were renewed daily (FP, FA). All samples were delivered to SGS labs (Guelph, ON, Canada) to determine average moisture content (%), pH, and nitrogen concentration (%) (Table 1). 

### 2.4. Push-Door Setup

**Figure 3:**
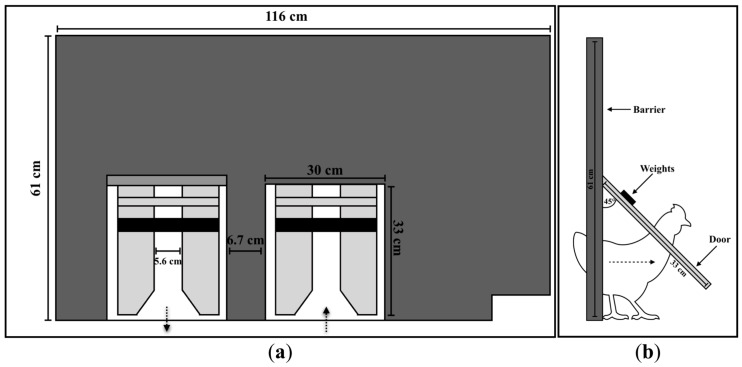
(**a**) Barrier layout with push doors viewed from chicks’ eye level. Each barrier contained two one-way push doors. Weights are shown as black bars that mount just above where the chicks’ heads would be when pushing the door. (**b**) The door rests on the chick’s shoulders as she pushes through the door. The maximum amount of weight the chick would feel would be when the door is at a 45° angle.

The T and H compartments of each pen were separated by a wooden barrier that contained two one-way polycarbonate push-doors. Lifting the doors to keep them open (0%) allowed the chicks to show their preference for resources without a challenge. To measure their motivation to access a treatment, we determined the median weight of the chicks in each pen and weighted the door to access T to 10%, 20%, and 30% of the chicks’ bodyweight—therefore presenting a challenging obstacle on a systematically varied schedule (Figure 3a). Once they moved into T, the chicks could return to H through another one-way door that was lifted (at 0% door weight) or unweighted (at 10%, 20%, and 30% door weights). Birds were habituated to this set-up for 7 days until experimental testing started at 22 days of age.

The maximum amount of weight that the chicks felt occurred when the doors were at a 45° angle and amounted to ≈50% of the actual weight of the door. This difference in calculation means that the same absolute door weight in this study could be used to represent a larger percent of body weight in another study. However, in performing this calculation, we believe our study more accurately reflects the degree of challenge imposed upon the chicks by more accurately representing the percentage of body weight that they pushed (Figure 3b).

### 2.5. Protocol

The birds were tested with each door weight and litter treatment combination according to a systematically varied schedule every day for 24 days, starting when chicks were 22 days old. Resources and door weights were changed every day. As such, each group of birds received all possible treatments of combined door weights and resources. When removing the previous day’s resources, SP and TSP litters were stored while fresh beddings (FP and FA) were discarded. When placing the new resource, T and H compartments were switched to prevent side bias.

Birds were individually weighed every third day to adjust the weight of the door to correspond to the median weight of the chicks in a pen. Standard health monitoring of the birds occurred on a daily basis. Additionally, all birds were assessed for footpad dermatitis at this time according to the 2009 Welfare Quality^®^ Consortium [40]. All chicks were restricted to H via barriers until shortly before video recording began. 

The cameras (Samsung SNO-5084R, Samsung Techwin Co., Gyeonggido, Korea) placed above each pen recorded daily from 12:30 to 22:30 and then from 06:30 to 09:00. To collect data on the birds’ time spent in T and the number of times the birds accessed T, we performed instantaneous scan sampling of individual birds’ positions in T and H once every half hour for 27 time points a day.

### 2.6. Statistical Analysis

Data were analyzed using SAS Studio (SAS Inst. Inc., Cary, NC, USA). Each chick’s time spent in T was calculated based on the number of time-points an individual was present in T out of a total observed time-points, while the number of visits to T was determined by counting the number of entrances to T. Maximum price paid was calculated as the mean maximum door weight pushed by all birds to access a resource. The percentage of time chicks spent in T, number of entrances to T, and maximum price paid to enter T were analyzed with a Gaussian distribution, and results are shown as LS means ± SE. The models were evaluated to ensure they met the assumptions of normally distributed residuals and homogeneity of variance, which were graphically inspected using QQ plots. Generalized linear mixed models (GLIMMIX) assessed the effect of treatment (feed, FP, FA, SP, TSP), door weight (0%, 10%, 20%, and 30%), and their interaction on time spent in T, number of entrances to T, and maximum price paid. Statistical significance was considered at *p* < 0.05 and tendencies are reported at 0.05 ≤ *p* ≤ 0.1.

## 3. Results

The chicks began the experiment at 22 days old, weighing an average of 0.62 ± 0.097 kg, and ended the experiment at 45 days old, weighing 1.87 ± 0.267 kg. One chick died for unknown reasons at 23 days old. Data garnered from when she was alive is included in the analysis. No other mortalities or clinical signs of disease were observed. Additionally, none of the chicks showed signs of footpad dermatitis during the experiment.

### 3.1. Motivational Index: Maximum Price Paid to Enter Treatment Compartment with Different Resources

**Figure 4:**
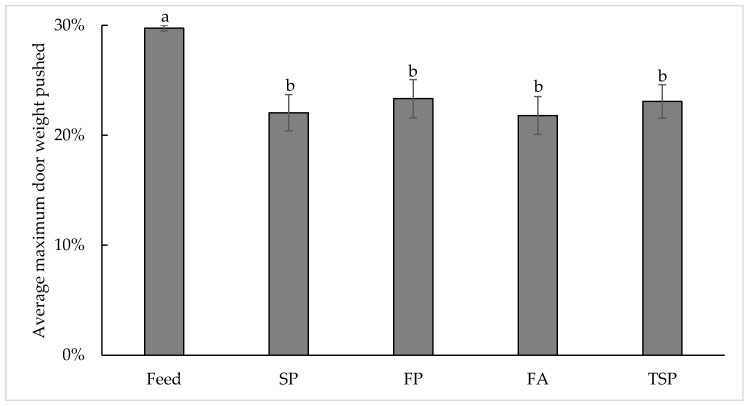
The average maximum weight pushed (0%, 10%, 20%, or 30% of chicks’ median pen body weight) to access resources in the treatment compartment (feed, soiled pine and spruce wood shavings (SP), fresh pine and spruce wood shavings (FP), fresh aspen wood shavings (FA), ammonia reductant treated soiled pine and spruce wood shavings (TSP)). Bars with different letters display significant differences (*p* < 0.05).

Resource significantly affected maximum price paid (F_477_ = 16.90, *p* < 0.0001), with chicks pushing a higher percentage of body weight on average to access feed (30% ± 0.3%) compared to FP (23% ± 1.7%; t_40_ = 3.65, *p* = 0.0042), TSP (23% ± 1.5%; t_40_ = 4.32, *p* = 0.0004), SP (22% ± 1.7%; t_40_ = 4.61, *p* = 0.0002), and FA (22% ± 1.7% t_40_ = 4.58, *p* = 0.0002. On average, chicks pushed the same maximum percentage of body weight to access all substrates (Figure 4).

### 3.2. The Effect of Increasing Cost on Visits to and Time Spent in the Treatment Compartment

#### 3.2.1. Number of Visits to the Treatment Compartment

**Figure 5:**
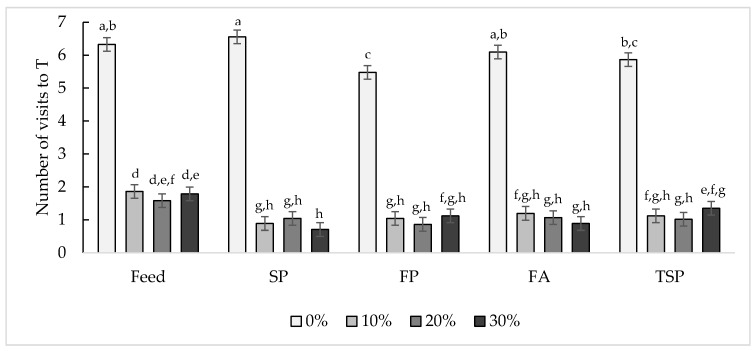
Effect of door weight (0%, 10%, 20%, 30%) and resource (feed, soiled pine and spruce wood shavings (SP), fresh pine and spruce wood shavings (FP), fresh aspen wood shavings (FA), ammonia reductant treated soiled pine and spruce wood shavings (TSP)) on the mean number of visits (±SEM) into the treatment compartment over a period of 13.5 h per day. Bars with different letters display significant differences (*p* < 0.05).

A significant interaction between door weight and resource was found for the number of visits to T (F_12,723_ = 2.40, *p* = 0.0048, Figure 5). In general, door weight affected the number of times chicks entered T (F_3723_ = 937.81, *p* < 0.0001), with chicks entering more often at 0% than 10% (t_723_ = 42.80, *p* < 0.0001), 20% (t_723_ = 43.75, *p* < 0.0001), and 30% (t_723_ = 43.825, *p* < 0.0001). The resource also affected the number of times chicks entered T (F_4723_ = 10.54, *p* < 0.0001) as chicks entered the feed treatment more often than FA (t_723_ = 4.56, *p* < 0.0001), FP (t_723_ = 6.05, *p* < 0.0001), SP (t_723_ = 4.66, *p* < 0.0001), and TSP (t_723_ = 4.36, *p* = 0.0001). However, no differences were observed in the number of visits to the different substrate resources (*p* > 0.05).

#### 3.2.2. Time Spent in the Treatment Compartment

**Figure 6:**
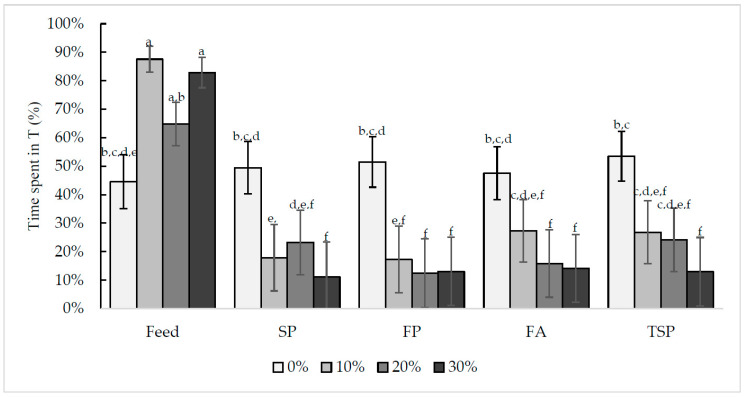
Effect of door weight (0%, 10%, 20%, or 30% of chicks’ body weight) and resource (feed, soiled pine and spruce wood shavings (SP), fresh pine and spruce wood shavings (FP), fresh aspen wood shavings (FA), ammonia reductant treated soiled pine and spruce wood shavings (TSP)) on the mean time spent (%) in the treatment (T) compartment for 13.5 h per day. Bars with different letters display significant differences (*p* < 0.05).

There was a significant interaction between resource and door weight (F_12,724_ = 8.41, *p* < 0.0001, Figure 6). The chicks spent equal amounts of time in all five resources when the doors were lifted (0% door weight). However, as door weight increased (10%, 20%, and 30%) the chicks spent less time in the substrate treatments and more time in T with the feed treatment (Figure 6). Additionally, the effect of door weight on time spent in T was the same for all substrates (F_3725_ = 18.23, *p* < 0.0001).

## 4. Discussion

This study used a two-compartment choice test to determine broiler chicks’ motivational strength for four different litter substrates (FP, FA, SP, TSP) based on their responses to increased access costs (push-doors weighing 0%, 10%, 20%, or 30% of a pen’s median chick body weight). Their motivation to access litter was then compared to the chicks’ motivation to access feed, the gold standard of comparison in motivation tests [32]. The chicks’ motivation was assessed based on the maximum price paid—or maximum weight pushed—to access the different resources. We predicted that the chicks would differentiate between the substrates in a rank order from most to least preferred: FA, TSP, FP, SP. We also predicted that increasing the cost to access the resources would reduce the number of chicks’ visits to the treatment compartment (T) as well as increase the time spent in T. Our findings indicate chicks worked hardest for feed and, contrary to our expectations, they did not pay a higher maximum price, spend more time on, or visit one substrate more than the other.

As predicted, broiler chicks paid the highest price for feed, showing feed is a physiological necessity [35] and that chicks, comparatively, are less motivated to work for bedding and litter (collectively referred to as substrates). These results also align with Dawkins [41], who found that adult laying hens worked more to access feed over a mix of sawdust and peat moss. The finding that broiler chicks are more motivated to access feed over dustbathing or foraging substrates is also consistent with food- or dustbathing-deprived laying hens, who more consistently chose feed in a Y-maze even when deprived of dustbathing substrates [42]. Like feed-deprived laying hens, broiler chicks’ disrupted satiety mechanisms [43]—and therefore genetically increased motivation to eat—makes feed an even stronger motivator [44] and may further drive a motivation to prioritize feeding over other behaviors. Moreover, broiler chicks’ excessive weight, accompanied by a possible shift in centre of gravity, makes activity tiring [45], which suggests they might require more motivation to overcome the push-doors to access a resource.

Against our predictions, the maximum price paid for the different substrates did not vary, indicating that the birds were equally motivated to access all substrates. This may be explained by the suitability and attractiveness of the substrates for different activities the bird wished to perform, such as foraging and dustbathing [46,47]. However, this study does not report how chicks spent their time on the different substrates and no definite statements can be made based on the current study. Nevertheless, young chicks are motivated to perform active behaviors (such as exploration, foraging, and dustbathing) and rest on substrates. Thus, it is possible that all the substrates were of equal value, being equally stimulating or aversive, or provided equal immediate experience for these behaviors. Additionally, we cannot exclude the possibility that chicks simply worked for additional space to explore [48]. Similarly, the daily changing of resources (once every 24 h) may have made all the substrates conditionally equally rewarding [49].

These results also suggest that chicks did not differentiate between different wood shavings (FA and FP) or litter treated with an ammonia reductant (TSP). Moreover, the chicks were not willing to pay a higher price for unsoiled over soiled substrates, and therefore did not avoid excreta. As previously stated, this could simply be the result of the chicks’ inability to identify any difference between the substrates. Conversely, since animals do not always work for things that benefit them [50], the chicks may have also simply chosen not to work for them. Yet, many factors could influence the chicks’ perception of soiled substrates. For example, compared to red jungle fowl, commercial chicks have been heavily selected for meat production [51], which may influence their behavior to avoid excreta. Additionally, these young birds lack maternal care and guidance, which may influence their avoidance of environments similarly to how mother hens influence their chicks’ avoidance and preference for foods [52]. However, the potential unsuitability of wood shavings for resting, dustbathing [47,53,54], and foraging [55,56] may also have reduced the birds’ motivation to access the beddings (FA and FP). Otherwise, the potential aversive aspects of some substrates may have been offset by increased suitability for performing rewarding behaviors, such that the birds responded equally to all of them. For example, broken-down, degraded soiled wood shavings (H litter, SP, and TSP) may be a better dustbathing substrate compared to fresh wood shavings (FA and FP). In particular, friable, used wood shavings tend to have a smaller particle size, and were found to be more stimulating and adequate for dustbathing in laying hens [57]. At the same time, other aspects of soiled wood shavings (like ammonia for H litter and SP, or acidity in TSP) may have made these substrates less preferable, counterbalancing the rewarding qualities of soiled wood shavings for dustbathing [24,25]. Similarly, the higher acidity (average pH of 4.9 ± 2.08) and reduced atmospheric ammonia levels (which is shown by an increase in percent nitrogen in the litter) in TSP litter may have cancelled each other out, rendering TSP no more or less appealing than the untreated H or SP litters. Thus, since it is possible that the birds found both the unsoiled beddings and soiled litters equally unfit, repeating this experiment with a preferred substrate, such as sand [47], may clarify these results.

At 0% door weight (lifted doors) chicks visited and spent time in all resources a similar amount. Therefore, in the absence of an obstacle, the results for number of visits and time spent suggest that the chicks had an equal relative preference for all resources. At this door weight, the chicks spent an equal amount of time in H and T as they moved between compartments five to seven times per 13.5 h observation period. In this case, the lack of door weight and the absence of footpad dermatitis on the chicks’ feet likely permitted more frequent movement between compartments. Moreover, the additional space offered by the second compartment (T) could be viewed as an enrichment, which may have led to higher activity levels that were beneficial for their health [58].

When door weights were applied (10%, 20%, or 30%), the chicks spent more time in H and visited T less, but spent more time in feed compared to the substrates. Therefore, as cost (door weight) increased, the chicks rescheduled their behavior to visit the feed treatment less and increase the duration of each visit. This result is similar to findings in mice and mink [59,60]. Moreover, the chicks’ response to increasing door weight suggests that feed outranks other resources for which rescheduling did not increase the duration of visits (time spent).

Similarly to Cooper and Mason [60], this effect was not observed with all resources, as increasing costs led the chicks to spend less time with the substrates. In fact, the chicks only spent about 10–30% of their time with the substrates when the doors were weighted. This may imply that the benefits of accessing the substrates declined rapidly with time once they were obtained. In other words, the lower amount of time spent on the substrates may reflect the amount of time needed to perform a rewarding behavior on that substrate [60]. Alternatively, if the chicks moved onto the substrates in T to obtain extra space or distance from conspecifics, then the number of visits and duration of stay in T may be more dependent on individuals. Regardless, this view implies that the chicks’ choice to move between compartments was primarily impacted by their motivation. However, Bokkers et al. [44] note that broilers are limited more by their physical abilities than motivation, so the chicks’ weight likely also impacted their choices, especially at 10%, 20%, and 30% door weights. Although weight is limited to its effect on broilers’ mobility, as the systematically varied schedule of the different treatments ensured that door weights and resources were not confounded with age or weight of the bird.

Therefore, the simple familiarity of the home litter compared to the other substrates may have influenced their decision to remain in H rather than overcome an obstacle. This is especially relevant to young chicks that may lack the foresight to understand how soiled litter may be detrimental (e.g., in terms of footpad health) or beneficial (e.g., in terms of possible benefits of social homocoprophagy) to their health. Thus, the findings of this research suggest that chickens may be incapable of assessing the long-term consequences of contact with soiled litter [11,12,13,16], or the potential adverse health effects of pine and spruce bedding [17,18,19,20,21]. Additionally, the chicks’ early exposure to increasingly soiled shavings during their first 2 weeks of life may have habituated them to soiled litter before the experimental habituation period. However, the birds’ equal motivation to access the substrates may also be the result of different litter conditions compared to normal farm litter conditions. In particular, the increased airflow of the setup combined with diligent, daily litter monitoring when changing litter may have limited the severity of moisture and ammonia build-up, and thus reduced the aversiveness of H and SP litters. Laboratory analysis of the litters in our study revealed a similar average moisture content for H and SP litters of approximately 29%, which is below the 30% moisture content found to increase the risk of footpad dermatitis in meat birds [61], a common problem in turkey production. Moreover, maximum ammonia production occurs at litter moistures of 37.4–51.1 [14], which are higher than the reported average moisture content of H and SP.

Preference tests themselves must also be rigorously designed to ensure that the animals’ response corresponds to the experimenter’s question. While the birds in this experiment were habituated to the substrates, and used a validated operand (pushing a door) [34,62] to access each resource, a number of other design factors present limitations to this experiment. It should be kept in mind that this set-up only allowed for testing of one experimental substrate compared to the home litter, and presenting multiple of these substrates at the same time could have influenced birds’ choices. Additionally, the ability of litter to affect air quality around it makes this study a comparison of environments, more so than variables, which makes the task of pin-pointing what exactly birds are motivated to access more difficult [32]. However, considering the open pen design and well-ventilated environment, this may be less of an issue in our study. Additionally, some preference tests opt to test individual animals [63] to more clearly observe individual preference; however, we opted to use groups of animals. While chickens moving simply to flock together could affect our findings, de Jong et al. [47] found that housing hens in isolation made learning the push-door task more difficult. More importantly, chickens are not commonly housed in isolation, so our choice to house in groups could more accurately predict chicks’ choices. However, this experiment is also limited by human perspective, as the few choices presented to the chicks limited what they could or could not be motivated to access. Moreover, interpretation of the chicks’ response to the resources is also limited by a human perspective that views a soiled environment as undesirable, and does not fully understand chickens’ motivations. Animals also have personalities, which means that their motivation to access different options may vary, leading some animals to explore or interact more with the operand amidst a relatively bare environment [64]. Therefore, animal owners and/or farmers should diligently manage litter conditions, as broiler chicks will not avoid soiled or potentially harmful substrates.

## 5. Conclusions

This experiment is the first study to assess the preference of broiler chicks for soiled or unsoiled substrates. In the experimental conditions provided, broiler chicks did not appear motivated to avoid soiled substrates. Instead, they showed an equal relative strength of preference for all the substrates. Additionally, chicks did not show a high demand for any of these litters compared to their demand for feed. Furthermore, these findings emphasize the duty of animal owners and/or farmers to diligently manage litter conditions for the birds’ health, as birds will not avoid soiled or potentially harmful litter. However, further work is needed to determine bird preferences for litter management practices under commercial conditions and investigate long-term effects of these practices on broiler health and welfare.

## Figures and Tables

**Table 1:** The average moisture content (%), pH, and nitrogen (%) concentration of litter samples taken from the home compartment, as well as the four treatment litters. Home litter and soiled shavings were sampled at the start and end of the experiment, while ammonia reductant shavings were sampled at the beginning and end of the projected chemical saturation point (7 days). As fresh pine and spruce and fresh aspen litters were renewed each day only one sample was taken from these litters.

Litter Type	Average Moisture (%)	Average pH	Average Nitrogen (%)
Home compartment litter	29.4 ± 19.93	6.4 ± 0.16	1.7 ± 0.29
Soiled pine and spruce wood shavings (SP)	29.9 ± 16.67	7.3 ± 0.23	1.7 ± 0.04
SP treated with an ammonia reductant (TSP)	20.0 ± 1.94	4.9 ± 2.08	1.8 ± 0.32
Fresh pine and spruce wood shavings (FP)	18.61	9.33	0.25
Fresh aspen wood shavings (FA)	5.57	5.26	0.34
